# Deficient approaches to human neuroimaging

**DOI:** 10.3389/fnhum.2014.00462

**Published:** 2014-07-01

**Authors:** Johannes Stelzer, Gabriele Lohmann, Karsten Mueller, Tilo Buschmann, Robert Turner

**Affiliations:** ^1^Department of Neurophysics, Max Planck Institute for Human Cognitive and Brain SciencesLeipzig, Germany; ^2^Danish Research Centre for Magnetic Resonance, Copenhagen University Hospital HvidovreHvidovre, Denmark; ^3^Department of Biomedical Magnetic Resonance, University Hospital TübingenTübingen, Germany; ^4^Magnetic Resonance Center, Max Planck Institute for Biological CyberneticsTübingen, Germany; ^5^Nuclear Magnetic Resonance Unit, Max Planck Institute for Human Cognitive and Brain SciencesLeipzig, Germany; ^6^Department of Diagnostics, Fraunhofer Institute for Cell Therapy and Immunology, Leipzig, Germany; ^7^Department of Physics, University of NottinghamNottingham, UK

**Keywords:** fMRI, cognitive neuroscience, brain mapping, functional localization, critical neuroscience

## Abstract

Functional magnetic resonance imaging (fMRI) is the workhorse of imaging-based human cognitive neuroscience. The use of fMRI is ever-increasing; within the last 4 years more fMRI studies have been published than in the previous 17 years. This large body of research has mainly focused on the functional localization of condition- or stimulus-dependent changes in the blood-oxygenation-level dependent signal. In recent years, however, many aspects of the commonly practiced analysis frameworks and methodologies have been critically reassessed. Here we summarize these critiques, providing an overview of the major conceptual and practical deficiencies in widely used brain-mapping approaches, and exemplify some of these issues by the use of imaging data and simulations. In particular, we discuss the inherent pitfalls and shortcomings of methodologies for statistical parametric mapping. Our critique emphasizes recent reports of excessively high numbers of both false positive and false negative findings in fMRI brain mapping. We outline our view regarding the broader scientific implications of these methodological considerations and briefly discuss possible solutions.

## INTRODUCTION

**FIGURE 1:**
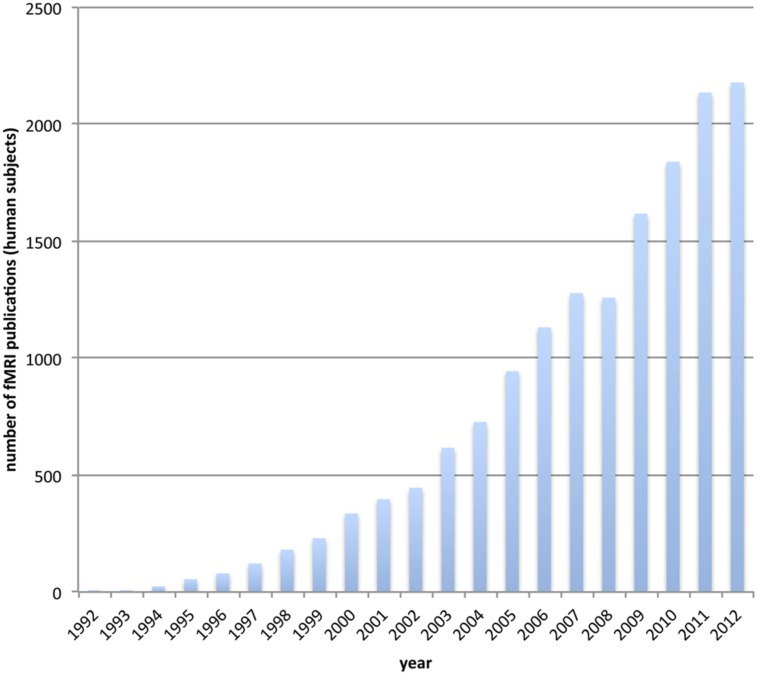
**The usage of functional MRI gets increasingly popular.** We depict the number of publications for each year that incorporate fMRI on human subjects. The data is based on a pubmed.org search (see appendix).

Functional magnetic resonance imaging (fMRI) has become the workhorse of human cognitive neuroscience. Brain scanners are now available at hundreds of research sites, including governmental institutions, universities and hospitals. The number of studies using fMRI for the investigation of human brain function is ever-increasing (see **Figure 1**). A simple search on pubmed.org (see appendix) reveals an accelerating yearly output, so that the number of studies published in the last 4 years (2009–2012) is about the same as the number published in the 17 years between 1992 (when it all started) and 2009.

Most of these studies are concerned with the localization of human brain function. The underlying goal of these efforts is obviously to characterize the relationship between brain structure and brain function, in a systematic fashion. For instance, studies may investigate which brain regions are recruited for music imagery, or for syntactic analysis of heard speech. Ultimately, the knowledge of such a structure to function relationship allows a better understanding of how the brain processes information. Furthermore, structure–function mapping allows insights into how and to what extent information processing is performed by functional subunits, and how these units interact in representing mental states or performing mental tasks. In summary, the most clearly definable goal of brain mapping is to establish links between neuronal substrates, their connections, and their functional relevance.

While many studies have investigated a wide range of human mental competences, often with striking findings, we question the validity of several underlying research methods for mapping brain function. In particular, we examine the serious pitfalls of the widely used methodologies that employ statistical parametric mapping and related concepts.

Several of the relevant issues have already been discussed over the last decade. Our aim here is to assemble them and consider their implications for scientific inference. We focus on aspects regarding data handling, omitting methodological aspects of fMRI data acquisition and neurophysiological interpretation which have been discussed elsewhere in detail (Logothetis, 2008). Our arguments do not apply solely to one particular method for constructing statistical maps of brain function, but rather pertain broadly. Our critique thus ranges from general linear models (GLM; Friston et al., 1995) to sophisticated information mapping methods using machine learning approaches (Haynes and Rees, 2006; Norman et al., 2006).

We argue that commonly applied brain mapping methods, implemented in a wide range of software packages that use some form of statistical parametric mapping, generally have a number of poorly explored inherent flaws, which when taken together may greatly reduce the adequacy and credibility of the resulting brain activation maps. There are two types of inherent flaws. Firstly, the reported findings may be largely incomplete, reflecting critically high levels of false negative attributions (also referred to as type II errors). In commonly used data analysis techniques, false negatives arise in several ways, discussed in detail below. It should be emphasized that each source of false negativity contributes additively to the overall number of false negatives; thus the effects accumulate. Secondly, brain function may be incorrectly attributed to specific anatomical locations that were not involved in the given task, for instance regions where there are no neurons. This kind of error is commonly known as false positivity (or type I error). While there have been systematic efforts to reduce the occurrence of false positives in neuroimaging (Lieberman and Cunningham, 2009), common brain mapping approaches enshrine further intrinsic and plentiful sources of false positivity, in a way which is rarely discussed.

Here first we summarize the general methodological framework^1^ used in most statistical parametric mapping studies. We then discuss the inherent flaws in this methodological framework. We discuss previous literature, as well as our own results. The discussion takes the perspective of false positivity and false negativity, ordered by their spatial scales from finest to coarsest. While these issues can also be described using receiver operating characteristic (ROC) plots (Fawcett, 2004), we discuss them here qualitatively. Finally, we conclude with a view of the resulting broader scientific implications. Drawing from a review of existing literature and original research, our conclusions sharply criticize currently accepted brain mapping epistemology.

## A GENERALIZED BRAIN MAPPING FRAMEWORK

**FIGURE 2:**
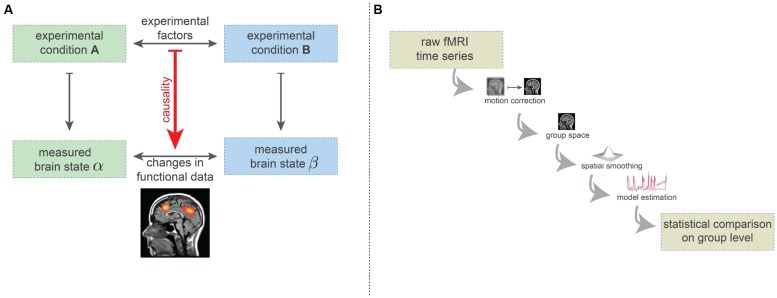
**(A)** Simplified basic experimental rationale underlying fMRI-based brain-mapping studies. Two (carefully chosen) experimental conditions elicit two distinct brain states. FMRI measurements are able to capture certain aspects of these brain states. The resulting images are compared statistically and this statistical difference is causally ascribed to the difference in terms of experimental factors. **(B)** Minimalistic preprocessing and data analysis pipelines. Preprocessing includes head-motion correction, transformation into a common coordinate system (e.g., Talairach or MNI space) and spatial smoothing. On this preprocessed data, model estimates are computed (here, we depicted the general linear model). The statistical comparison across the group usually is carried out on basis of the model estimates (e.g., contrasts) and incorporates a correction for the multiple comparisons problem.

Here we describe the simplified logic of brain mapping experiments. The vast majority of brain-mapping studies implement or build on this basic design (Carp, 2012b). In the simplest form, such experiments consist of two experimental conditions (see **Figure 2A**). The experimental conditions may, for instance, be the visual presentation of two different orientations of a grating, or the passive listening to grammatically correct and incorrect sentences. Importantly, the type and implementation of the conditions must be carefully chosen: while the desired experimental factors differentiating the two conditions need to be maximally isolated, the influence of other factors (e.g., extrinsic variables) should be minimized or at least quantified. While enriched experimental designs are often recommended, such as factorial or parametric designs or designs that explore the interactions of the experimental conditions, the basic procedure is the same—effectively to subtract mean voxel-wise amplitudes of the MRI intensity in order to estimate how much more activity occurs in specific regions of the brain for particular combinations of task conditions.

It is agreed that each experimental condition gives rise to a different brain state, in the sense of a spatio-temporal pattern of neuronal activity which is generally assumed to be somewhat stationary during the condition. Using neuroimaging techniques, certain aspects of these brain states can be measured (Logothetis and Wandell, 2004). The statistical factors differentiating the two measures of brain states are assumed to result from the difference between the two experimental conditions (**Figure 2A**). For instance, certain regions may show difference in the fMRI measurements *because* of the different visual grating orientations, or syntactical violations in language processing.

To differentiate the two measured brain states in a statistically acceptable way, various preprocessing and analysis methods are used. Many such methods have been proposed, but even with the most popular methods the number of plausible processing pipelines is about as great as the number of studies (Carp, 2012a). In **Figure 2B**, we display the minimal set of preprocessing and analysis steps used in the vast majority of studies. Firstly, subject head motion is corrected using retrospective realignment methods. Next, the individual fMRI data are normalized^2^ into a common coordinate space [e.g., MNI or Talairach space (Chau and McIntosh, 2005)], allowing comparison at the group level.

As a final preprocessing step, spatial smoothing procedures are applied which spatially blur the fMRI data. The preprocessed data are then analyzed using a model, such as the GLM (Friston et al., 1995). Here the quality of fit between a generative model and the actual data is computed. The GLM model is generated by convolving the time course of the experimental conditions (block design or event-related design) with a hemodynamic response function, which is considered to be identical for each brain voxel. Finally, a statistical comparison is carried out at the group level, testing for voxel-wise differences in the model parameters (e.g., the degree of fit of the response model). The resulting whole-brain statistical maps are then presented in a thresholded fashion, implementing a correction for multiple comparisons (i.e., correcting for the large number of statistical tests being carried out). Often the thresholding includes the assumption that connectedness increases the significance of brain voxels. All in all, this results in the widely known images of *“blobs”* of brain activity.

## SMALL SPATIAL SCALES IN THE BLIND SPOT

Typically, fMRI acquisitions are performed with an isotropic resolution of about three millimetres. At first glance it would appear that the *effective* resolution used for brain activation mapping is identical to the resolution of acquisition, in other words that activations typically only a few millimetres across can be resolved. However, preprocessing procedures applied on the raw fMRI data effectively *compromise* the effective resolution that is available for structure–function mapping. Depending on the methods employed, resolution can be lost by as much as a factor of 50 or even 100 (i.e., the smallest resolvable unit is in the order of magnitude of 50–100 voxels of the original acquisition). In the following paragraphs we describe the data preprocessing procedures that lead to this net reduction of resolution, and discuss what this implies scientifically.

### SPATIAL SMOOTHING

In most fMRI studies which use the previously introduced brain-mapping framework, Gaussian spatial smoothing is applied to the data as a preprocessing step (Carp, 2012b). After smoothing, each voxel contains a mix of its own signal and the weighted signal of surrounding voxels. The full width at half maximum (FWHM) of the smoothing kernel determines the contribution of surrounding voxels to the voxel of interest; larger kernel sizes give greater contributions from neighboring voxels. This smoothing procedure was proposed at a time when the only available method for functional brain imaging in humans was positron emission tomography (PET).

Smoothing was needed:

(i) to enhance the signal-to-noise ratio (SNR) by effectively averaging data across several adjacent voxels
(ii) to allow statistical inference using the theory of Random Gaussian Fields
(iii) to enable averaging across the spatially normalized brains of a subject group

At this point it should be highlighted that the sole justification for each of the above points is pragmatic usefulness. From a biophysical point of view, there is no first-principle reason that requires averaging the blood-oxygenation-level dependent (BOLD) signal over *space*.

In practice, spatial smoothing appears to improve the statistical sensitivity, as higher statistical scores are achieved when including smoothing. However, spatial smoothing brings severe side effects.

**FIGURE 3:**
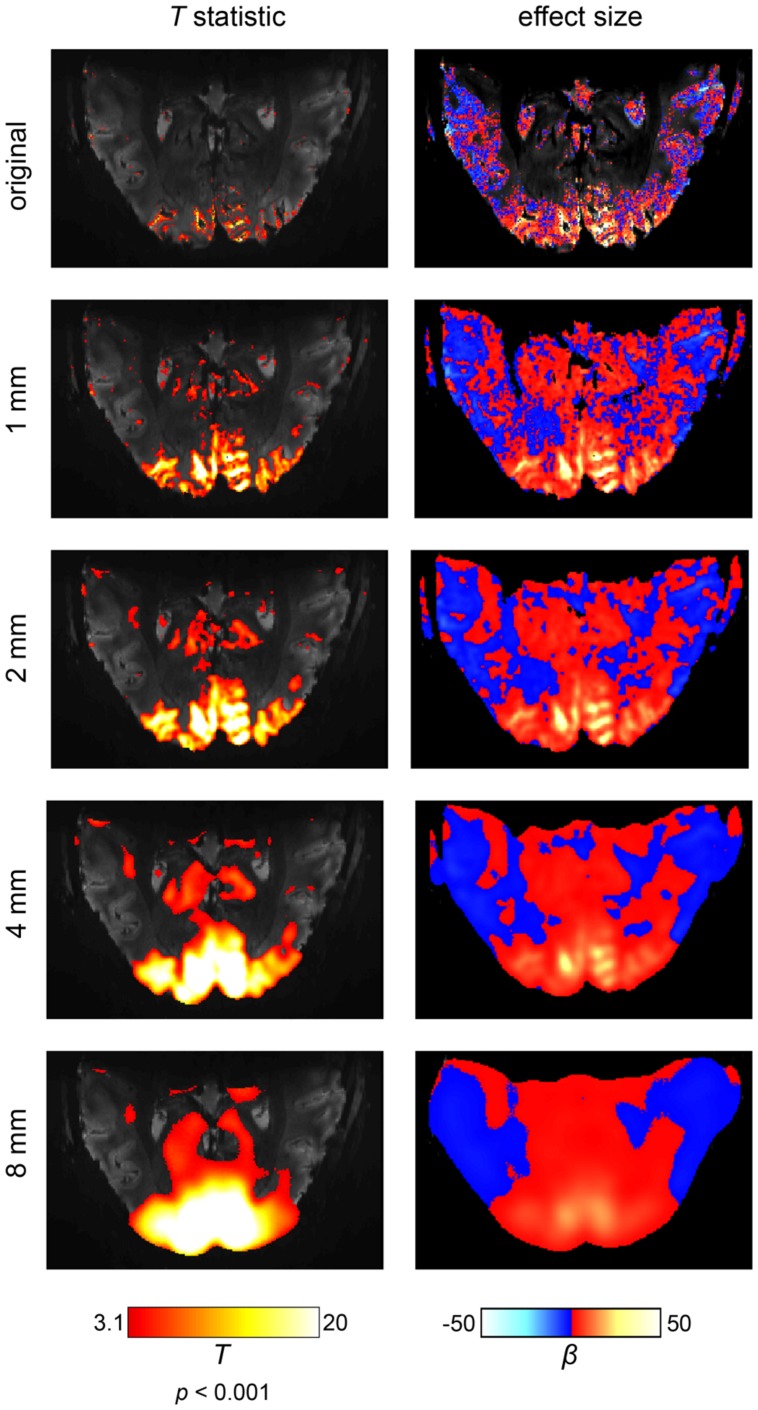
**The effects of spatial smoothing illustrated on an ultra-high field fMRI data set at a field strength of 7 Tesla and an isotropic resolution of 0.65 mm.** The scanning paradigm comprised a visual checkerboard stimulation (see appendix) and the analysis was based on a simple general linear model. In the left column, the results are displayed in terms of a *t*-statistic (i.e., significance of activations). In the right column, the corresponding effect sizes are shown (i.e., amplitude of activations). We depicted the original results and four levels of spatial smoothing. When smoothing is omitted, fine-grained activation patterns are visible on the cortical surface (i.e., within gray matter regions). While spatial smoothing increases the statistical significance of the results, both the effect size and spatial accuracy of the results are drastically reduced. Noteworthy, the intrinsic SNR would be increased if a larger voxel size was used or more repetitions were carried out. As result, a larger number of voxels would be labeled active.

The first side effect of spatial smoothing is an incorrect estimation of the true spatial extent of brain activations (Sacchet and Knutson, 2013). This effect becomes especially apparent in ultra-high resolution fMRI at 7T (Heidemann et al., 2012), as shown in **Figure 3**. It can be readily observed that separate and, most critically, *distinct* activations progressively merge together, dilating into a smaller number of larger activations. Spatial smoothing thus drastically distorts the extent and location of true activations. In particular, voxels that can never produce a real BOLD activation (e.g., because they lie within white matter or cerebrospinal fluid) may receive signals from its surroundings and appear active. Thus, these voxels erroneously display activation, induced solely by the spatial smoothing procedure. Furthermore, spatial smoothing may combine adjacent but distinct activations into one single activation (Geissler et al., 2005). The peak of such a combined activation, however, may be located in a region which never exhibited signal. For example, as depicted in **Figure 3**, even applying a rather mild 1 mm smoothing kernel can create apparent “activations” in anatomically impossible regions. Thus, spatial smoothing increases the numbers of false positive voxels, since spatial smoothing is likely to produce spurious activity in voxels that never originally contained relevant signal. The apparent spatial extent is driven mostly by the somewhat arbitrary choice of the smoothing kernel, so that cluster size and voxel counts, quite often used in data analysis, have very little biological meaning.

However, the opposite case (namely many false *negatives*) can also result from smoothing, particularly when there are:

(i) isolated signals of a limited spatial extent
(ii) low-intensity signals (possibly of larger spatial extent) near the non-active tissue.

In each situation, spatial smoothing will *decrease* the original signal from the voxels considered. At the same time, neighboring non-activated voxels will contribute perturbing noise. All in all, this decreased effective SNR results in false negativity for either of the above situations, a failure in the detection of true effects and signals. This has far-reaching consequences: spatial smoothing can make it practically *impossible* to detect activations of small extent or small amplitude, even when these deviate enough from some baseline to be considered significant if analyzed using more powerful statistical methods. Arguably, spatial smoothing can provide strikingly misleading interpretations of human brain function, as the results are strongly biased towards the appearance of large-scale activations which may be biologically implausible.

**FIGURE 4:**
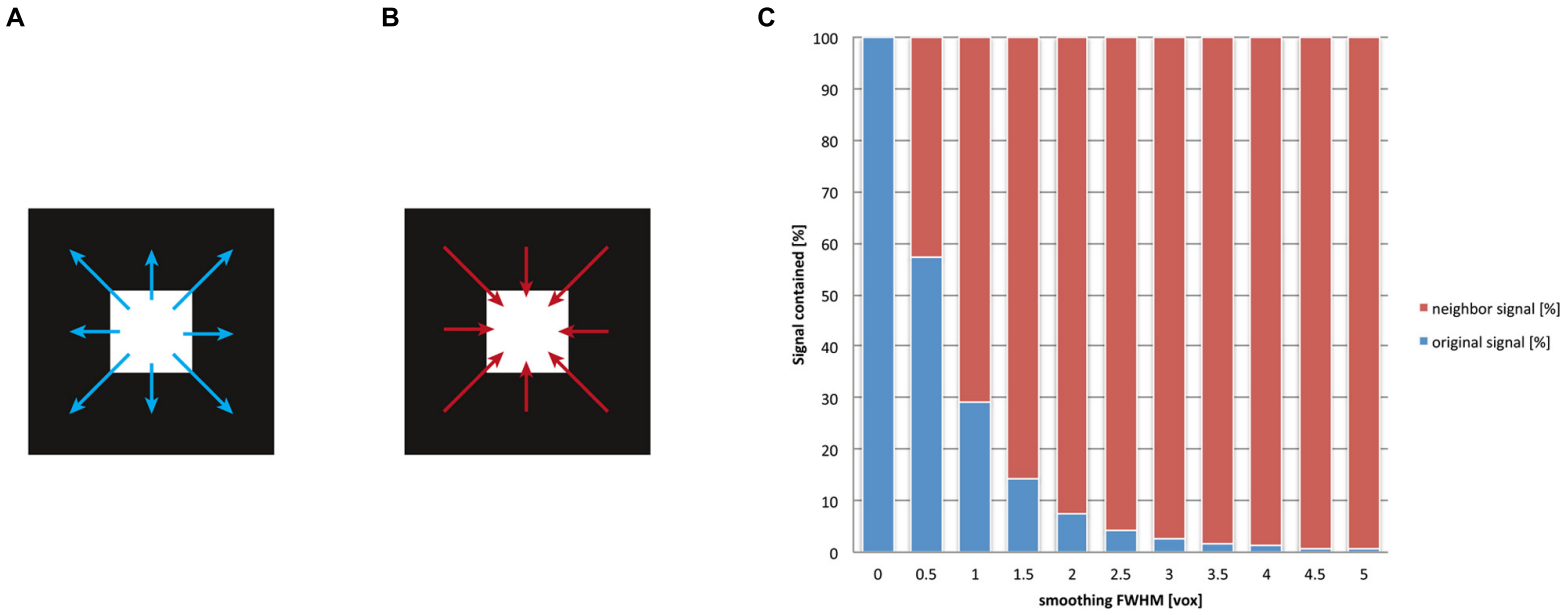
**After the application of spatial smoothing, the signal of a given voxel is a mix between its original signal and the weighted average of its surroundings.** Smoothing implies that for any given voxel **(A)** the signal is “washed” into its neighborhood and **(B)** that signal of its surroundings is washed into this voxel. **(C)** We display the ratio of this mix, that is, how much of the original signal remains in the voxel versus how much signal of the neighborhood is washed in. We computed this mix for different smoothing kernel sizes (given in the dimension of voxels, for details see appendix). The blue bars represent the original (internal) signal of a voxel; the red bars illustrate the fraction of the (external) signal that stems from the neighborhood of the voxel. It is visible that already for relatively small smoothing kernels (e.g., two voxels, corresponding to a FWHM of 6 mm given 3 mm voxels), more than 90% of the signal does not correspond any more to the original signal at any given voxel but stems from the voxel’s neighborhood. This implies a severe loss of spatial precision for functional localization.

As we have discussed previously, after spatial smoothing, the signal of a voxel is effectively a *mix* between the original signal of that voxel and the weighted signal of the neighborhood. The ratio of this mix depends on the smoothing kernel. For another example of the smoothing’s drastic influence on localizability, we depicted the ratio of the mix between local and neighborhood signal in **Figure 4** for multiple levels of smoothing. Evidently, even for rather small values for the FWHM (one voxel, e.g., FWHM = 3 mm for a voxel size of 3 mm) the contribution of the voxel’s neighborhood is *twice* as big as the contribution from the (original) signal at this location. Traditionally the size of the smoothing kernel is set between 8 and 10 mm for whole-brain studies, which returns the most favorable results from a pragmatic point of view (Mikl et al., 2008). More recently, however, kernel sizes of 6 mm have become more customary. For imaging certain structures (e.g., subcortical nuclei), it is normal to use even smaller smoothing kernels. However, it should be stressed that the theory of Gaussian random fields provides reliable estimates of statistical significance only when smoothing kernels have at least twice the voxel size (Worsley and Friston, 1995). Given smoothing kernels of such dimensions, more than 90% of the post-smoothing signal at any given location does not stem from the original location but from voxels in its neighborhood. Notably, this calculation holds for the *overall* smoothness, which should not be equated with the size of the smoothing kernel applied within the preprocessing: in fact fMRI images may already exhibit an *intrinsic* smoothness, additive to the smoothing procedure. The intrinsic smoothness may originate both from biophysical properties of the BOLD signal (Malonek and Grinvald, 1996; Kriegeskorte et al., 2010) and image interpolations that take place in previous preprocessing steps (Kamitani and Sawahata, 2010), such as motion correction or spatial normalization to the standard space. Hence our depiction in **Figure 4** is rather conservative, as the effective^3^ smoothness generally is larger than the FWHM provided for spatial smoothing. Note that the so-called “draining vein” effect (Turner, 2002), which can cause mislocalization of BOLD signal, does not in general increase the spatial smoothness, because pial veins are generally much smaller in diameter than the size of the fMRI voxel. In any case, this mislocalization only becomes severe in the unusual situation where only a few veins drain a large area of activated cortex. Here the spurious BOLD signal can often be identified as a linear structure following veins that are visible on the structural image.

Together these issues indicate that brain activation maps produced with spatial smoothing should be interpreted with great caution regarding the localization of brain activity. This is particularly true for larger smoothing kernels between sizes of 8 and 10 mm. Yet, however, despite these issues, virtually no authors presently include a discussion regarding the underlying spatial precision in the localization of brain function. The same holds for the occasional extreme overestimation of spatial extent, as shown in **Figure 3**.

Surface-based smoothing (Jo et al., 2007) mitigates some of these issues. In particular, the spatial accuracy and sensitivity are improved. For a given kernel width, the influence of a voxel’s neighborhood is smaller and less signal is blurred out, thus surface-based smoothing is gentler as compared to volume-based procedures. Furthermore, the pitfall of smoothing across sulcal banks is avoided. Besides these improvements, however, spatial inaccuracies within the cortical plane remain.

Multivariate pattern analysis methods (Carlson et al., 2003; Kamitani and Tong, 2005; Haxby et al., 2011) generally do not require spatial smoothing of the fMRI data. However it should be noted that in certain cases the *analysis method itself* systematically introduces spatial smoothness, a loss of spatial precision. The multivariate searchlight technique is an example of such methods (Kriegeskorte et al., 2006). It has been shown that this method may lead to severe spatial inaccuracies (Viswanathan et al., 2012; Etzel et al., 2013), such as apparent activations in white matter. In particular, the searchlight method exaggerates the spatial extent of informative areas, which is especially unwelcome for ultra-high field fMRI (Stelzer et al., 2014).

### CLUSTER-BASED STATISTICAL INFERENCE

The number of statistical tests carried out in typical fMRI experiments is huge, as an *individual* statistical test is performed for *each* voxel. Assuming an isotropic resolution of three millimetres and whole-brain coverage at a field strength of 3T, the numbers of voxels and thus individual tests is roughly 50,000. This necessitates a *correction* for the large number of tests (known as correction for multiple comparisons). Without a proper multiple comparisons correction, many voxels may erroneously appear to be statistically significant (solely due to the sheer number of tests). Traditional multiple comparisons strategies, such as the Bonferroni correction (Miller, 1981) are far too conservative (and yield false negatives). The main reason for this lack of power is that the significance of each voxel is tested separately, treating voxels as *independent* from each other. However, neighboring voxels can exhibit spatial correlation, especially after spatial smoothing, and thus there is dependency structure between the tests. Cluster-based statistics explicitly utilize these spatial dependencies. Clusters^4^ are generally defined as spatially contiguous groups of voxels, all of which surpass a fixed statistical threshold of activation. The underlying rationale behind cluster-size inference states that it is more unlikely to find *two neighboring* voxels both surpassing a statistical threshold than *one single* voxel surpassing the same threshold (Forman et al., 1995). Hence the unit of interest, on which the test statistic is applied, is not each voxel, but a spatially contiguous *region*(Heller et al., 2006). The crucial step in cluster-size inference is thus correcting for multiple comparisons on the level of *clusters* rather than *voxels*. This greatly reduces the number of tests that need to be performed: instead of considering 50,000 statistical tests (on each voxel), only a few dozen clusters require a correction test. The statistical tests at the cluster level are then commonly corrected using false-discovery rate (FDR) methods (Benjamini and Heller, 2007; Chumbley et al., 2010). This decreased severity of the multiple comparisons problem may be the main reason for the broad dissemination of cluster-based methods, as these have been shown to be more powerful than voxel-based tests (Hayasaka and Nichols, 2003). As an alternative to regarding the spatial extent alone, unified statistical frameworks have been proposed which also take into account peak heights (Worsley et al., 1996). It should be noted, however, that the random field framework and its derivatives critically rely on smoothed data, and require strong and hard-to-verify assumptions (Genovese et al., 2002).

Cluster-size thresholding usually results in a minimum cluster size that is deemed statistically significant; larger cluster sizes surpass and smaller clusters are rejected. However, the gain in overall statistical power comes at a price, which, although being obvious, is nevertheless heavy: all clusters that are smaller than the minimum cluster size are sifted out. In other words, the power of detecting small-scale activations is greatly diminished. In fact, the power to detect activations smaller than the minimum size drops to zero. Hence methods explicitly considering the connectedness of voxels are a potential source of false negativity.

**FIGURE 5:**
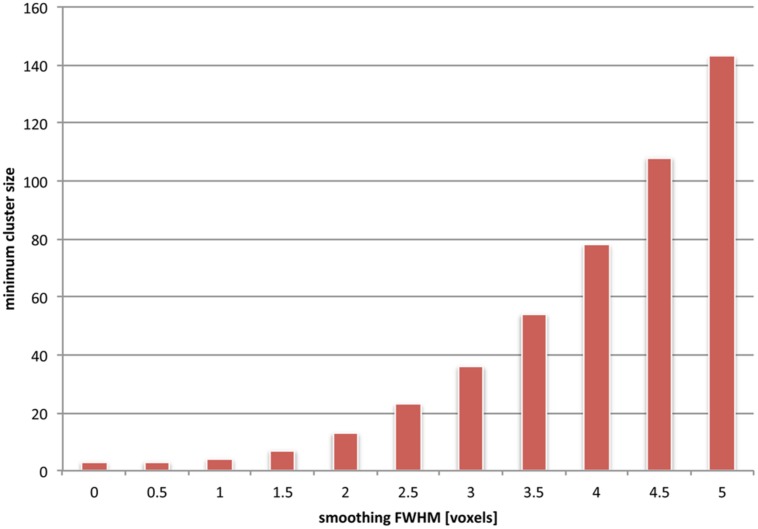
**The effects of combined spatial smoothing and cluster-based statistics are additive.** We used simulations to depict the minimum cluster size which is determined as significant for various levels of smoothness (see appendix). Clusters of smaller extent than this minimum size fail to reach significance and are effectively sieved out. For instance, if an overall smoothness of 4 voxels is assumed in the underlying images, only clusters that are larger than about 80 voxels are taken into consideration (i.e., are corresponding to an uncorrected cluster *p*-value < 0.05). It should be noted, however, that this *uncorrected* cluster *p*-value is still subject to a multiple comparisons correction; hence depending on the number of overall clusters the final minimum cluster size may actually be considerably larger than the value we depict here.

The frequency of false negative voxels at small spatial scales induced by cluster-size thresholding on the one hand, and spatial smoothing on the other, is unfortunately *additive*. To visualize this, we used simulations (see **Figure 5**) to compute the dependence on underlying image smoothness of the minimum cluster size. The minimum cluster size corresponded to a probability *p* < 0.05 of occurrence. It is clear that the minimum cluster size depends monotonically on increasing smoothness. Moreover, the dependency is non-linear; given our simulations, doubling the smoothness effectively quadruples the minimum cluster size. This may result from multiple separated clusters merging into fewer, larger ones. In the worst case, clusters can be declared significant, which span multiple distinct anatomical regions (Woo et al., 2014), and are thus hard to interpret in a scientifically meaningful way.

Hence it can be concluded that spatial smoothing and cluster size inference act together, in regard firstly to generating misleading information about spatial location and extent (false positive attributions), and secondly to increasing the number of false negative voxels at small spatial scale. When both methods are combined (which is common practice), it becomes virtually impossible to detect spatial activations smaller than a certain size. Thus, due to this combination of methodologies, small activations are *a priori* excluded from contributing to the spatial representation of mental states. Noteworthy, what we designate here as *“small spatial scale”*(ranging from 50 to 100 voxels) in reality reflects on the order of 50–100 million cortical neurons.

## GROUP ANALYSIS–MAPPING THE EFFECTIVE OVERLAP

Group-level analysis has become the standard practice in cognitive neuroscience for localizing brain functions. The main theoretical motivation to study brain function at the group level is to identify *universal* processes of human brain function and cognition (Friston et al., 1999). The identification of universal mechanisms within a given population has been very fruitful for fields of biology, particularly in physiology. Consider for instance the study of an internal organ of the human body: Following the assumption of a universal mechanism, it is possible to abstract *general features* and principles of functionality that are *shared* amongst the human population. Using these abstracted features, the role of the organ, its constituents and the interaction with the overall system can be investigated and understood in a general sense. Furthermore, it is possible to delineate and characterize inter-individual differences, which may themselves be linked to genetic or environmental factors.

Aside from this theoretical motivation, in the domain of neuroimaging there are further rather pragmatic reasons to carry out studies at the group level. Most importantly, if perfect coregistration of the corresponding areas of subjects’ brains could be guaranteed, the *power* of statistical tests (i.e., the ability to detect neuronal activations), would be *substantially* higher at the group level than at the single-subject level. For somatosensory stimulation, where the location of the relevant brain areas is known to be well conserved across individuals, the power has been shown to increase for larger number of subjects tested within a study (Desmond and Glover, 2002).

A major reason for this gain in statistical power with group size is the high level of variance typically found in experimental neuroimaging data. Critically, this variance is interpreted as nuisance or noise (Horn et al., 2008). It is commonly assumed that by averaging over subjects the noise-related part of the signal is diminished, allowing true effects to emerge.

However, the apparent gain in statistical power for group-level studies gives rise to rather severe drawbacks, in the form of both false negative and false positive attributions. These drawbacks are a direct consequence of the (implicit) assumption of *universality* mentioned before. In neuroimaging, this assumption can be formulated more sharply as: *the spatio-temporal dynamics of brain functions have a high degree of uniformity within a population.* This implies that, at their core, brain dynamics (and their governing activation patterns) are assumed to be largely *similar* across subjects, both temporally and spatially. Critically, deviations from this universal “fingerprint” of a given brain function are then ascribed to the* noisy character* of the underlying brain dynamics.

Although there is excellent reason to attribute universality within and even across species at microscopic levels of brain function, namely at the level of single neurons, there is no good *a priori* reason for attributing universality to more complex brain functions, taking place at the level of interacting large-scale networks. What if there is often little uniformity in the neuronal representation of human brain function at higher levels (thought and cognition), which involve networks of billions of neurons? What impact would non-uniformity have on the validity of standard group statistics, which fundamentally rely on the uniformity assumption?

From a *structural* point of view, the practice of warping individual brains into a standard space imposes limitations here. In particular, cortical areas often exhibit sharp boundaries (Clarke and Miklossy, 1990; Schleicher et al., 1999; Geyer et al., 2011), while on the other hand, the morphology and folding patterns may differ strikingly across subjects (Rademacher et al., 2001; Fischl et al., 2008). Current methods for warping the functional data into the standard volumetric space (e.g., MNI space) cannot take this into account. Such methods imply an irreversible loss of information in regards to the brain architecture, effectively conserving only large-scale features (Turner, 2013). The same arguments holds for surface-based methods (Fischl et al., 1999), as long as they are based the geometric similarity of the folding patterns. It should be noted that surface-based registration of myeloarchitectonic features, as revealed in T1 maps of the cortex (Tardif et al., 2013; Van Essen and Glasser, 2014) promises to provide far more precise registration of functionally congruent brain areas.

**FIGURE 6:**
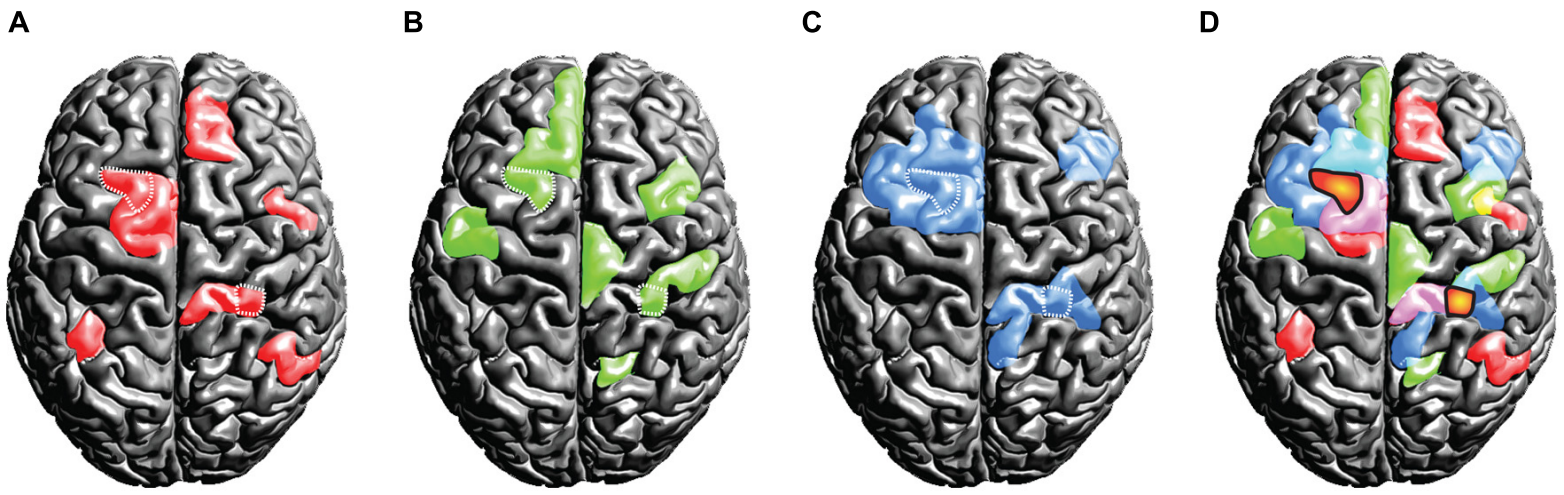
**Thought experiment considering the activation patterns for three subjects **(A,B,C)**.** The individual (ground truth) activations of the three subjects are displayed in the colors red, green, and blue. Critically, we assume the true activations to be variable across the subjects. If standard group statistical procedures then are applied on this scenario, only the *effective overlap* of the subjects is revealed **(D)**. We display this effective overlap in an orange *“blob-like”* tone in **(D)**, for the sake of illustration we marked the overlap also in the individual subject patterns **(A,B,C)** using white dots. Under the assumption of high inter-individual variance, this illustration shows the fallacy of spatial group statistics: for *none* of the subjects the overlap regions were sufficient for representing the brain state, as each subject relied on the involvement of further regions.

From a *functional* point of view, another set of problems arises. Following the assumption of universality, mixed- and random effects group-level methods in neuroimaging (Penny et al., 2003; Mumford and Nichols, 2006) treat overlapping activations shared across subjects as true activation. Critically, activations that are *not shared* across the group and only emerge in a small subset of the tested population are implicitly considered noise. However, if the assumption of uniformity is not true and subjects exhibit *fundamental* differences in their spatio-temporal representations of brain function, it would be premature to label voxels activated only in a *subset* of the subjects as noise. We have outlined this scenario in a thought experiment that assesses some brain function *Y* invoked by task *X* in a group of three subjects (). Crucially, as this is a thought experiment, we know the ground-truth involvement of brain areas that the experimental task *X* causes for each subject (displayed in red, green and blue). When commonly practiced group statistics are employed, the group activation pattern will be subject to substantial erosion (as compared to the ground-truth activations for each single subject): only at locations where all subjects feature an involvement, the group-level inference indicates a reliable activation (as marked by the orange blobs in **Figure 6D**). Much in the sense of an *“effective overlap,”* only these common areas are revealed as the final result of a group analysis, while all other activations are considered noise and are discarded. Consequently, *only* the overlapping region will be ascribed the brain function *Y* of carrying out the specific task (the brain region is often then termed as the “center of *Y*”). However, in light of our thought experiment (which assumes a violation of the uniformity assumption) the overlap areas were not sufficient to carry out the ascribed brain function *Y* on their own. From a conceptual point of view, there is no a priori reason (other than the assumption of uniformity) why overlapping regions should have a *special role* in task performance. In point of fact each subject may critically rely on an orchestrated interplay of *all* brain areas that had been involved.

The above considerations reveal the potential fallacy of group-level statistics on a rather theoretical premise. In particular, the thought experiment demonstrated the hypothetical weakness of the assumption of *uniformity of brain function* across a population. In summary, the aim of the thought experiment was to portray the consequences of a naïve group-level analysis if large-scale uniformity does not hold. Critically, both registration errors (when normalizing individual data into a common stereotactic space) and true underlying functional differences may contribute to the violation of the uniformity assumption.

From an empirical point of view, the validity of the uniformity assumption (on a large spatial scale) remains contentious. On the one side, evidence does suggest a certain degree of uniformity. For instance it is possible to train pattern recognition algorithms to distinguish two brain-states in one subject and then successfully apply the learned model on another subject (Shinkareva et al., 2008; Clithero et al., 2011; Kaplan and Meyer, 2012). Such a cross-subject classification can only be possible if the spatio-temporal patterns of brain activation are quite similar across different subjects. These findings thus indicate that at least some aspects of brain organization indeed show a degree of generalization or uniformity within a given population.

**FIGURE 7:**
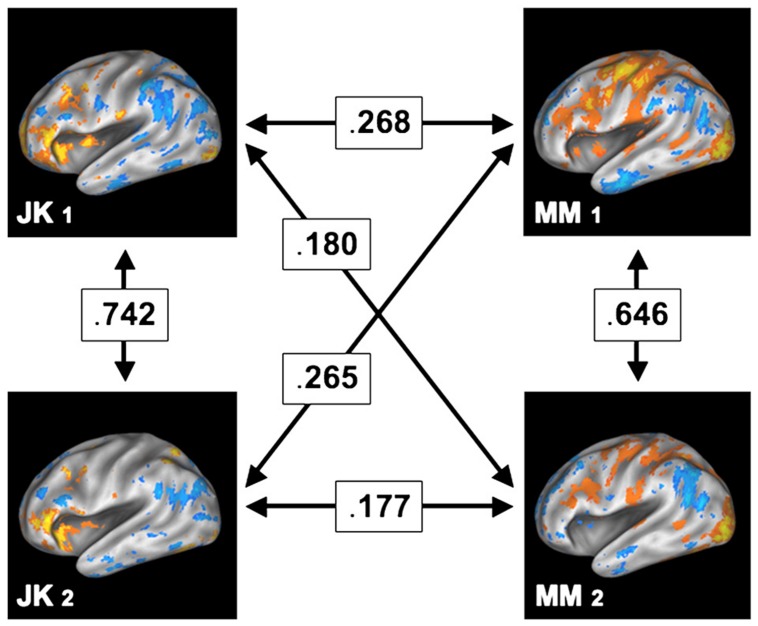
**Test and retest study, where two participants (JK and MM) were scanned on two separate sessions, held months apart.** The arrows in between the participants and sessions denote the cross-correlation between the (uncorrected) patterns of brain activity, thus indicating their degree of similarity. While the spatial patterns remain relatively similar within a subject (across both sessions), the similarity across subjects is comparably low. Reprint with permission from Miller et al. (2012).

In contrast to such findings, *test and retest studies* where the same subjects are being scanned repeatedly reveal a strikingly different picture. For instance, Miller and colleagues (Miller et al., 2009, 2012) have shown that *within* the same subject the encoding brain areas for a memory retrieval task remained highly similar between two scanning sessions separated by months. However, in contrast, the degree of similarity *across* subjects was substantially lower, as shown in **Figure 7**, reprinted from their paper. Among other conclusions from this finding is the direct refutation of the ascription of noise to voxels outside of the “effective overlap,” that is, those voxels which are not consistently active across subjects. It is most unlikely that these voxels would show activity in another fully independent measurement several months later if they were due to spurious activity. Consequently, the regions not shared between subjects (which erode in group-level analysis) in all likelihood exhibit functional relevance. Most importantly, this functional relevance cannot be easily generalized within a population, as other subjects did not feature involvement of the same areas. Hence, ultimately, this empirical evidence contradicts the naïve assumption of large-scale universal brain function.

In summary, the assumption of universal brain function has been neither validated nor fully disproved. While there is undeniably some inter-subject consistency, different subjects may have substantial discrepancies in terms of anatomy and, in addition, *utilize* their brain in quite distinctive ways. It appears conceivable that this *diversity* of brain representations depends on the level of brain function. Lower-level brain function may feature less variability across subjects than high-level mental functions (e.g., executive functions, cognitive control, social behavior). Indeed, the inter-subject variance of deeper cortical folds (which are the first to form in human development) has been shown to be lower than the variance of shallower ones (Lohmann et al., 2008; Fischl, 2013). On the other hand, this does not imply that low-level sensory areas cannot vary substantially across subjects. For instance, the surface area of early visual areas may vary by a factor larger than two across individuals (Dougherty et al., 2003). Sources for inter-subject variability at a range of spatial scales include genetic makeup, epigenetic differences, and neuroanatomical variability due to general life experiences (Horn et al., 2008). It is vitally important to recognize that brain mechanisms, whether actual or modeled, can only operate in *individual* living brains. Averaging brain function across subjects thus creates an abstraction of brain function that is assumed representative for the population. However, as we have argued, the assumptions for this abstraction are not met and hence such abstractions are inappropriate for mechanistic explanation.

This argument suggests that maps of brain activations are likely incomplete, and miss important elements. In other words, common group-analysis methods can result in many *false negative* findings (which may be spatially widely distributed) because true individual activations may not be adequately captured in group maps. This fully justifies the terminology of false negatives, defined as the failure to detect true effects which may be critical for brain functionality. Furthermore, the functional role of the overlap region is unclear, as the overall areas involved with specific functions may substantially differ across subjects. Thus it may be difficult to interpret the role of areas which show good overlap after group statistical analysis.

Group-level inference may also create false positive voxels (i.e., an erroneous ascription of activity). In earlier examples we described group-level statistics as a method that delineates the “effective overlap” of activity within a given population (see **Figure 6**). This analogy, in the mathematical sense, implies that within this perimeter *all subjects* exhibit an involvement of the respective brain area. This leads in turn to the question whether such group-level activations can be observed in situations where only a *proportion* of the subjects feature a sufficiently strong activation while the remaining subjects *do not* activate this brain area.

To investigate this empirically, we simulated a statistical assessment of group-level activity, with a random-effects analysis, carried out on 20 (virtual) subjects. Importantly, the subject group was *heterogeneous* in its composition and consisted of a subgroup of *responders* (exhibiting an effect) and a subgroup of *non-responders* (where the effect was absent). Using our simulation, we varied the fraction of non-responders within the group, ranging from a pure responder group to a full non-responder group. Furthermore, we also varied the size of the effect (within the responder subgroup) to investigate its dependency on the group-level inference.

**FIGURE 8:**
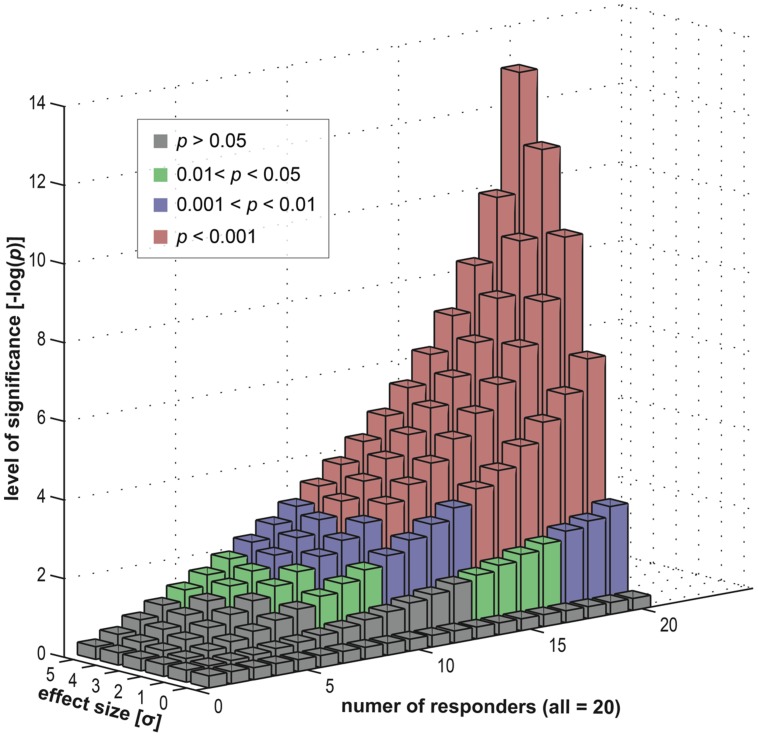
**Data simulation investigating the effects of a *heterogeneous* subject group (in terms of responders and non-responders).** We computed group statistics (*t*-based) for one single location using a group of 20 “virtual” subjects (see appendix). This group consisted of two subgroups, firstly responders and secondly non-responders. The former were sampled from an effect distribution (normal distribution with an offset) and the latter from a null distribution (normal distribution). We varied the composition of the group (i.e., the number of responders versus the number of non-responders) and also the size of the effect (the offset). For each level (20 levels of group composition and six levels of effect sizes) we repeatedly computed a *t*-based one-sample random-effects analysis, as used in common group level inference. We displayed the resulting statistical significance levels in different colors: green for mild significance (0.01 < *p* < 0.05), blue for moderate significance (0.001 < *p* < 0.01) and red for high significance (*p* < 0.001).

Results of this simulation in **Figure 8** show that a specific activation is not necessarily co-localized even in a majority of subjects to achieve a highly significant result at the group level. Depending on the underlying effect size, high significance may be reached even if only half the subjects show an effect. This implies that the other half of the subjects showed no involvement of this brain area. Nevertheless, on the group level, typical fMRI studies ascribe an effect at such a given position in the *entire* subject group – and hence in the population from which the group is selected. The concept of an *“effective overlap”* is thus imprecise. What the typical analysis reveals is only the *“effective overlap of a subset of subjects that show a sufficiently large effect size.”* When subject groups are heterogeneous – and without looking at individual subject data, this heterogeneity remains unknown–standard strategies for group-level inference indeed generate false positive voxels, erroneously generalizing a localized effect in some subjects to the entire group.

The explicit consideration of inter-individual differences, on the other hand, offers interesting further possibilities for research. Given that there are differences across subjects, it is worthwhile to compute comparisons between individual activation patterns (Thirion et al., 2007). Furthermore, it is highly interesting to systematically investigate the origin of such variations in brain activity within a population (MacDonald et al., 2006; Horn et al., 2008; Kherif et al., 2009; Miller et al., 2012), which may interestingly reflect inter-individual cognitive styles and strategies.

Yet another problem arises in relation to spatial smoothing and averaging across subjects. This relates to the irreversible loss of information that this entails, which also has a bearing on the likelihood of false positives. In theory, the process of Gaussian smoothing can be reversed using deconvolution with the same kernel (Kamitani and Sawahata, 2010). Once smoothed data have been averaged across subjects, however, the original detail of any given subject is no longer retrievable by a deconvolution operation, especially if the smoothing kernel is much larger than the voxel size (as is the custom) and data are somewhat noisy. Combining smoothing and averaging then becomes an irreversible, effectively non-linear, operation. Furthermore, such methods are coupled with the extreme non-linearity of thresholding at a specified level of statistical significance, which transforms a continuously varying *t*-score into a binary variable defining whether or not a given voxel is significant. Taken together, it is easy to see that the final extracted parameters such as location and spatial extent may have a very limited relationship with the original data.

In summary, we propose that inter-subject variability should be considered in most neuroimaging studies (i.e., in addition to intra-subject effects). Studying such variations offers rich insights and allows drawing a much more complete and realistic picture of human brain function, from which models of neural mechanisms can be postulated. Studies ignoring such inter-subject variations should be interpreted with far greater caution, as they may present many false positive and false negative findings.

## MODEL AND RELIABILITY ISSUES

Statistical parametric maps of neuroimaging data aim to visualize the involvement of brain regions in a given task, which is hypothesized to be associated with specific postulated brain mechanisms. Typically, this is achieved by fitting a response function to the neuroimaging data. The quality of the fit between this model and the experimental data is then evaluated using various statistical measures. For instance, in the case of the GLM, a response function is constructed by convolving a generic hemodynamic response function with the onset timing of each task or condition. The parameters giving the best fit between the generated response function and the experimental neuroimaging data are then statistically further evaluated.

Crucially, the data explained by the overall model is considered signal, while the remainder is considered noise. This practice, however, raises a few issues. In particular, the generative models may only explain a very small fraction of the signal’s variance. However, instead of reporting the quality of the fit in terms of explained variance or effect size, results are often reported exclusively on the basis of the probability of the rejection of the null hypothesis. Low *p*-values (i.e., high t or* Z*-values), however, should not be confused with large effect sizes or an adequate fit between the model and the data – in fact highly significant models can go hand in hand with a non-existent model fit (Lohmann et al., 2012). Hence the ascription of noise (often termed *physiological noise*) to the portion of data that remains unexplained may be premature. Much of this noise has indeed been shown (Bianciardi et al., 2009) to arise from spontaneous fluctuations of neural activity. For instance, Lohmann and colleagues (Lohmann et al., 2010) used fMRI data from several language and non-language experiments and regressed out all model contributions derived from a GLM approach. Using only this residual “noise” data, it remained possible to identify temporally correlated networks that were present exclusively for the language experiments, networks that are suggested to form part of a general framework in the language domain. Importantly, the time-locked experimental variance (which was regressed out) only accounted for a comparably *minor* fraction of the variance in the empirical data.

The results of neuroimaging studies thus critically depend on the choice of analysis model. It is more than likely that simplistic analysis methods, such as GLM-based methods, are unable to reveal the full picture. In particular, such massively univariate methods consider neuronal communication processes only indirectly as they mainly evaluate whether significant activity occurs at any specified voxel. Since the major functional role of neurons is to transmit co-ordinated activity to separate places in the brain, more sophisticated analysis methods which consider simultaneously the BOLD signal at multiple voxels may thus be better suited to analyze human brain function (Lohmann et al., 2013b). It is vital to recognize, however, that not all multivariate models considering neuronal communication (e.g., in terms of effective connectivity) are free of problems. In particular, dynamic causal modeling (DCM) has been shown to be especially problematic due to several critical methodological flaws (Lohmann et al., 2012, 2013a; Friston et al., 2013). Taken together these issues severely limit the scientific validity of the conclusions that can be drawn when using DCM.

The analysis model, however, is not the only factor yielding diverging results. The particular choice, order and parameters of the preprocessing steps (e.g., spatial normalization, motion correction etc.) have also been shown to have a considerable impact on the resulting activation maps (Carp, 2012a). The amplitude and location of the peak activation have been shown to be especially unstable and subject to considerable variability. This issue becomes especially problematic for two reasons: firstly, many authors do not describe design and analysis decisions in sufficient detail (Carp, 2012b). Secondly, this variability of outcome can easily tempt researchers to implement various different sets of preprocessing pipelines (in a trial-and-error fashion) and to selectively report the most favorable results (Carp, 2012a).

Furthermore, the extent of brain activation can be severely underestimated using traditional scanning paradigms. In a recent ground-breaking study, Gonzalez-Castillo et al. (2012) demonstrated that traditional fMRI paradigms may suffer from inadequate power in detecting true effects. Instead of scanning a large number of subjects for a relatively short time period, the commonly used procedure, they scanned very few subjects for a long cumulative duration, over many scanning sessions. In total, an unconventional number of 100 runs per subject were acquired (achieved by concatenation over about ten scanning sessions per subject). For the experiment, the subjects performed a simple visual discrimination task. The authors then systematically investigated how the resulting activation maps (derived by a GLM analysis without spatial smoothing) depended on the number of runs included in the analysis. This was achieved by inputting only a subset of the runs into the analysis, and then averaging over this subset. When including a conventional number of runs (i.e., about five to ten), the resulting activation maps were sparse. This is well in line with previous imaging studies, which indicate that only a small fraction of all voxels becomes active for such basic tasks. The situation changed however, more runs were included. Generally, the number of activated voxels increased monotonically when more runs were taken into the analysis. Interestingly, when all of the 100 runs were included into the analysis, about 70% to 90% of all voxels were labeled as active. The number of significant voxels furthermore depended on the response model used for the GLM analysis; the number of significant voxels was higher when allowing more unconventional response shapes (e.g., in the form of deactivations). Furthermore, the number of active voxels failed to converge within the tested regime, for either of the different GLM response models.

The study thus indicates possible *brain-wide modulations* of BOLD activity in response to tasks that are not of random nature. This result raises the fundamental issue that virtually every fMRI study may have overlooked the involvement of many brain areas, simply because there was not sufficient power (i.e., scans) available. In other words, the study suggests that there is a substantial false negativity problem, inherent to all spatial scales. The second important issue regards the interpretation of results; if it is true that even for a simple task the entire brain becomes involved, then the dichotomy of labeling brain areas as active or inactive is no longer meaningful and scientifically relevant. To put it pointedly, these results may herald the end of *qualitative* activation-based neuroimaging.

## CONCLUSION

In our article we have outlined the most important sources of *false positive* and *false negative attributions* that are inherent to widely used techniques for human brain mapping (as shown in **Figure 2**). We have argued that the quantity of false attributions incurred by these techniques is unacceptably large, and thus provides a misleading impression of human brain function. This, in turn, may lead to quite unrealistic models of brain mechanisms, and severely limit the validity of the scientific conclusions that can be drawn from brain mapping studies. The intrinsic unreliability of traditional methods that involve spatial smoothing, warping to a template and averaging may render the process of scientific theory and hypothesis testing inherently problematic.

In particular, the *qualitative* nature of the inference process may limit reliability and validity of brain-mapping studies. On one hand, the location and extent of the resulting thresholded activations (“blobs”) depends sensitively on the parameters and order of preprocessing and analysis procedures (such as smoothing, spatial normalization and statistical thresholding). On the other hand, the extent of activation maps strikingly depends on the available power of signal detection, as impressively demonstrated by the recent findings of Gonzalez-Castillo et al. (2012) that show that the number of activated voxels may – under optimized conditions – cover almost the *entire brain*. All in all this brings into question the adequacy of qualitative brain mapping: What can we usefully learn from the binary labeling of brain areas as active or inactive, if the entire brain may be involved in the representation of even the simplest tasks and associated functions?

Moreover, even the notion of an *abstract brain*^5^ is beset by problems. Such an abstract brain arises from generalizing from individual brains to a “group brain” representing group brain activity. The resulting findings and proposed mechanisms may never be adequately reflected in the *individual* brain, the only place where specific neurophysiological mechanisms can actually operate. Rather than being considered as a source of nuisance variables, the individual brain and its own inherent dynamics should be regarded as providing a *gold standard* for the investigation of functional mechanisms. The acid test of any proposed mechanism should be: Does it work in an individual brain?

### IMPLICATIONS ON THE EPISTEMOLOGICAL LEVEL

Above we have summarized our criticisms at the level of a single study viewed in isolation. In the following, we discuss the implications of our critique on a broader level of scientific inference.

Within the discussion section of neuroimaging papers, the supra-threshold group-level findings (“blobs”) of the respective studies generally are put into perspective with previous literature. This corpus of literature usually has examined either similar brain functions or found similar brain regions to be involved.^6^ In other words, neuroscientific inferences and conclusions for the interpretation of the present findings are drawn from a *network* of *related evidence* regarding structure–function mappings. Unfortunately, however, the overwhelming majority of the studies comprising this network of references suffer from exactly the same methodological weaknesses as those we have described here. These weaknesses are consequently *carried over* onto a broader level of scientific inference. “Garbage in, garbage out” is the dictum of data analysis—and the custom of spatial smoothing has the unfortunate effect of transforming good data into garbage. This may severely spoil any qualitative theory or meta-analysis that compiles and integrates structure–function relationships across studies. Above all, the lack of resemblance of the averaged data to the results from any of the individual subjects may severely hamper the testing of hypotheses and theories.

From an epistemological perspective further rather general issues arise. On this broader level, the *qualitative* argumentation and inference process for establishing structure–function relationships is prone to problems: as the number of fMRI experiments is ever increasing (as shown in **Figure 1**), it is frankly impossible for researchers to link and discuss the relation of their present findings with the entire relevant corpus of literature (namely studies concerning similar brain functions or revealing similar activations). Consequently, the reasoning can never be based on the full picture, but rather on a sparse selection of articles. Ultimately, there may be a temptation for researches to include studies which rather support their particular qualitative theory, as opposed to findings that cannot be integrated satisfactorily into their relevant explanatory framework. Large-scale data mining projects, collecting and making searchable results from neuroimaging studies, are likely to mitigate the dangers of such practices. Most critically, such data mining should not be based on the reported results of individual studies [such as the Neurosynth project (Yarkoni et al., 2011)], because their results suffer from the same plethora of false positive and negative voxels, and mislocalization of activation maxima. To avoid these pitfalls, data mining efforts should be based on the actual *raw* fMRI data.

### TERMINOLOGY OF PSYCHOLOGY

Apart from the methodological issues concerning fMRI-based brain mapping experiments, it is worthwhile mentioning controversies regarding the *terminology* of cognitive neuroscience. This primarily considers the *factors* that differentiate the experimental conditions (see **Figure 2A**) and their interpretation. Unfortunately, many widely used terms are ambiguous (Poldrack et al., 2011), as miscellaneous definitions are used in the literature. However, it cannot be emphasized strongly enough how important an objective terminology is when it comes to adequate scientific reasoning. Consider for instance terms from neuroscience such as synapse, neuron, action potential, cortical column, gyrus and sulcus (Turner, 2012). It is easy to define such terms objectively, thus allowing qualified scientists to identify and investigate the object of research. This is, however, not always the case for terms used in *cognitive* neuroscience: consider for instance terms such as perception, consciousness, attention and altruism. These terms are often vaguely defined, if at all. Thus, the employed terminology is often beyond objective scientific definability (Turner, 2012). Curiously enough, there are even cases where the terminology of brain-mapping studies closely resembles Gall’s phrenology (Poldrack, 2010). Finally, the terminology of cognitive neuroscience may depend on the cultural background and the current Zeitgeist. It thus remains unclear whether a consensus ontology is achievable at all.

### BEYOND BASIC BRAIN MAPPING

There have been encouraging recent developments that may help to avoid some of the most egregious misuses of fMRI and MRI data. We describe what we consider to be the most promising perspectives.

A desirable trend is to *publish entire data sets*, including the fMRI raw data and behavioral paradigms (Poldrack et al., 2013). Critically, this would allow other researchers to re-analyze data with the help of new methodological developments and furthermore help to assess the reliability and stability of the results. Additionally, data-mining efforts can be based on such databases.

Changing the order in which the statistical analysis of fMRI data is carried out may help the interpretability of the data. In principle, what should be averaged across subjects are the model parameters extracted from analysis of each subject (Turner, 2013). This directly allows one to examine inter-subject variation, and hence to decide whether the model is worth pursuing further. In particular, it would be possible to test whether the proposed mechanisms actually take place in the individual brain. In practice, conjunction analysis (Heller et al., 2007) may be of great usage here, as the functional variability across subjects is taken explicitly into account. Crucially, such analysis allows reporting effects on the group level but at the same time how frequently the effects actually are found in the *individual* brains. In the same breath cross-validation procedures should be mentioned, as these allow testing for the *reliability* of effects across subjects. The procedure may reduce the type I error rate while at the same time maintaining high levels of sensitivity.

This requires, however, a spatially precise normalization to a group template. An important step towards this can be achieved by explicitly taking into account the individual myeloarchitecture (Tardif et al., 2013). Ultimately, single-subject cortical parcellation enabled by *in vivo* observation of myeloarchitecture may offer the most reliable results (Geyer et al., 2011; Bazin et al., 2014); however, this would not result in a spatial *map* of brain activity.

Preprocessing of the data should omit spatial smoothing. Thus, multivariate approaches are a natural choice for data analysis, as no smoothing is required here (Carlson et al., 2003; Kamitani and Tong, 2005; Haxby et al., 2011). Furthermore, integrating information from many brain locations may be more sensitive for identifying brain mechanisms as compared to univariate approaches (Norman et al., 2006). Particular mention is to be made in regards to the ongoing rapid development of multivariate machine-learning methods, although Nishimoto et al. (2011) have already shown that sequences of stimuli can be reconstructed from the measured fMRI data. Network modeling may come to be a key strategy for identifying relevant functional structures in the human brain (Sporns, 2013). Effectively, such network-based approaches may help to characterize the brain on its own terms as a complex dynamic system (Lohmann et al., 2013b). Simulation of brain dynamics using biophysically realistic simulations (Deco et al., 2008; Markram et al., 2011; Gerstner et al., 2012) offers promise for the identification and understanding of brain mechanisms, in particular by bridging all spatial scales.

## Conflict of Interest Statement

The Associate Editor Dr. Daniel S. Margulies declares that, despite having previously collaborated with the authors, the review process was handled objectively. The authors declare that the research was conducted in the absence of any commercial or financial relationships that could be construed as a potential conflict of interest.

## Footnotes

1 A more thorough description of the fMRI framework can be found here (Ramsey, 2002).

2 As a side note, the transformation into standard space itself is not without controversy due inter-individual differences in brain anatomy (Brett et al., 2002; Carp, 2012b).

3 The effective smoothness consists of the intrinsic (biophysical) smoothness and the explicit smoothing applied as preprocessing step.

4 The term “cluster” is used historically in fMRI research, however in general such spatial structures are referred to as “connected components.” More generally and particularly in the context of unsupervised learning, clusters are referred to as groups of similar objects.

5 Such “abstract brains” are typically also the object of Dynamic Causal modeling.

6 Noteworthy, this form of *reverse inference* is controversial, as the functional relevance of brain activations is not necessarily comparable across studies (Poldrack, 2006).

## APPENDIX

### PUBMED.ORG SEARCH STRING (Figure 1)

We used the identical search as used by Carp (Carp, 2012b) string on pubmed.org for counting the number of human fMRI studies per year (substituting *YYYY* with the year of interest).

“YYYY”[Date - Publication] AND (fMRI[Title/Abstract] OR functional magnetic resonance imaging[Title/Abstract] OR functional MRI[Title/Abstract]) AND brain[Title/Abstract] AND humans[MeSH Terms].

### 7T fMRI (Figure 3)

We used experimental data from an ultrahigh-field experiment at 7T, recorded by Heidemann et al. (2012) using a voxel size of 0.65 mm isotropic (the detailed scanner paradigm can be found at the original publication). The functional paradigm consisted of a simple visual stimulation, realized by blocks of 28 s stimulus-on and 28 s stimulus-off. In total 15 epochs were used and the total acquisition time was 14 min. The stimulus-on blocks consisted of a flickering black and white checkerboard (at 8 Hz), while for stimulus-off, an isoluminant gray background was presented. One single representative subject was selected for the presentation in our results. We applied five levels of smoothing (no smoothing, 1 mm, 2 mm, 4 mm and 8 mm) and afterwards fitted a standard GLM model using LIPSIA (Lohmann et al., 2001) on the data. For the *t*-maps, we applied a statistical threshold of *p* < 0.001, without incorporating a multiple comparisons correction.

### SIGNAL MIXING DUE TO SMOOTHING (Figure 4)

We created a volume of the dimension 101 × 101 × 101 (voxel size = 3 mm) filled with zeros. The central voxel then was manually set to the value of 1. Next we applied spatial smoothing on this original volume, using different values for the size of the smoothing kernel, ranging from 0 to 5 voxels in steps for 0.5 voxels (i.e., 0–15 mm in steps of 1.5 mm). The value of the center voxel indicated how much of the original signal remained at this position. The sum over the entire volume discarding the central voxel then represents the fraction of the signal spreading outside the center voxel.

### MINIMUM CLUSTER SIZES (Figure 5)

We constructed 500 original volumes by sampling each voxel’s value from a Normal distribution *N*(0,1). The volumes were then smoothed using different smoothing kernel sizes. The kernel sizes (FWHM) ranged from 0 to 5 voxels in steps of 0.5 voxels. After this, the volumes were rescaled using a global factor so that the variance was equal to 1. The volumes were binarized by the application of a *z*-threshold of 2.33 (i.e., voxel *i* was set to 0 if its value *x_i_* < = 2.33 and set to 1 if *x_i_* > 2.33). Next we counted the resulting cluster sizes *s* of the binary images and computed their histogram *H*. After normalizing this histogram so that $\sum_{s=2}^{\infty }$ H(s) = 1, we determined the smallest cluster size *u* for which $\sum_{s=2}^{u}$ H(s) > 0.95 (i.e., cluster sizes with a probability of occurrence smaller than 5%). The procedure was repeated for each level of smoothness.

### HETEROGENEOUS SUBJECT GROUP SIMULATION (Figure 8)

For each level of considered effect size *s* (*s* = 0, 1,…,5) we sampled *n* values representing non-responders (*n* = 0, 1,…,20) from a Normal distribution *N*(0,1) and *r* values representing responders (*r* = 20 – *n*) from a Normal distribution *N*(*s*,1) with an offset *s*(representing the effect). We computed a *t*-test against zero using this combined sample of 20 values. For each value of *n* and *s*, the procedure was repeated 500 times and the resulting *p*-values were averaged.
